# Voltage-Controlled Magnetic Anisotropy in Fe_1−*x*_Co_*x*_/Pd/MgO system

**DOI:** 10.1038/s41598-018-28445-3

**Published:** 2018-07-09

**Authors:** Amit Kumar Shukla, Minori Goto, Xiandong Xu, Kohei Nawaoka, Joko Suwardy, Tadakatsu Ohkubo, Kazuhiro Hono, Shinji Miwa, Yoshishige Suzuki

**Affiliations:** 1 Graduate School of Engineering Science, Osaka University, Toyonaka, Osaka 560-8531 Japan; 2 Center for Spintronics Research Network (CSRN), Osaka University, Toyonaka, Osaka 560-8531 Japan; 3 Center for Materials research by Information Integration (CMI2), Research and Services Division of Materials Data and Integrated System (MaDIS), National Institute for Materials Science (NIMS), Tsukuba, Ibaraki 305–0047 Japan; 4 Research Center for Magnetic and Spintronic Materials, National Institute for Materials Science (NIMS), Tsukuba, Ibaraki 305-0047 Japan

## Abstract

Voltage-controlled magnetic anisotropy (VCMA) in an epitaxially grown Fe/Fe_1−*x*_Co_*x*_/Pd/MgO system was investigated using spin-wave spectroscopy. The spin-wave resonant frequency linearly depended on the bias-voltage. The resonant-frequency shift increased with the Co fraction in Fe_1−*x*_Co_*x*_/Pd. We achieved a VCMA of approximately 250 fJ/Vm at the Co/Pd/MgO region.

## Introduction

The control of magnetism using voltage attracts a significant attention in the field of spintronics. It is of significance in high-density low-power-consumption memory and ultrathin ferromagnetic films owing to the unique physical properties. Various experimental and theoretical studies in this field have been reported including studies on voltage-controlled magnetic anisotropy (VCMA)^1–6^, Curie temperature^7,8^ exchange bias^9^, Dzyaloshinskii–Moriya interaction^10^ and exchange interactions^11–13^. In particular, high-frequency magnetization switching has been utilized for a new class of VCMA-driven memory devices^14–17^.

The VCMA effect is induced by an accumulation of charge at the interface of ferromagnetic materials owing to an applied electric field. The accumulated charge screens the electric field in the region within a few monatomic layers of the interface. Therefore, the interface of ferromagnetic materials is of importance for the VCMA. The microscopic origin of the VCMA effect at the interface of ferromagnetic materials can be understood as follows. For 3*d*-ferromagnetic materials such as Co, it has been experimentally shown that electric-field-induced changes of the orbital magnetic moment predominantly contribute to the VCMA effect^18^. Moreover, for 5*d*-materials with proximity-induced spin-polarization, such as Pt, the magnetic dipole *T*_z_ term is of importance for the VCMA effect^19^. This term corresponds to the electric quadrupole in the atoms. This study indicates that the occupancy of the interfacial *d*-band in a ferromagnetic material is correlated with the VCMA effect. However, to the best of our knowledge, the dependence of the VCMA and interfacial anisotropy energy on the occupancy of *d*-band electron orbitals of a ferromagnetic material has not been studied.

In this study, we demonstrated VCMA and interfacial anisotropy field in the Fe_1−*x*_Co_*x*_/Pd/MgO system. The occupancy of the *d*-band electron orbitals in the Fe_1−*x*_Co_*x*_ alloy can be controlled with the Co fraction *x*. As the use of Pd at the Fe(Co) interface increases the VCMA^20–22^ a 0.2-nm-thick Pd layer (corresponding to one atomic layer of Pd) was introduced at the Fe_1−*x*_Co_*x*_/MgO interface.

In the first part of this letter, we characterize the crystal and layered structure of V/Fe/Fe_1−*x*_Co_*x*_/Pd/MgO using reflection high-energy electron diffraction (RHEED) and high-angle annular-dark-field scanning transmission electron microscopy (HAADF-STEM), and determine the position of the Co and Pd layers using energy-dispersive X-ray spectroscopy (EDS). In the second part, we investigate the fourfold crystal anisotropy field, interfacial anisotropy field, and VCMA in the Fe_1−*x*_Co_*x*_/Pd/MgO system.

**Figure 1:**
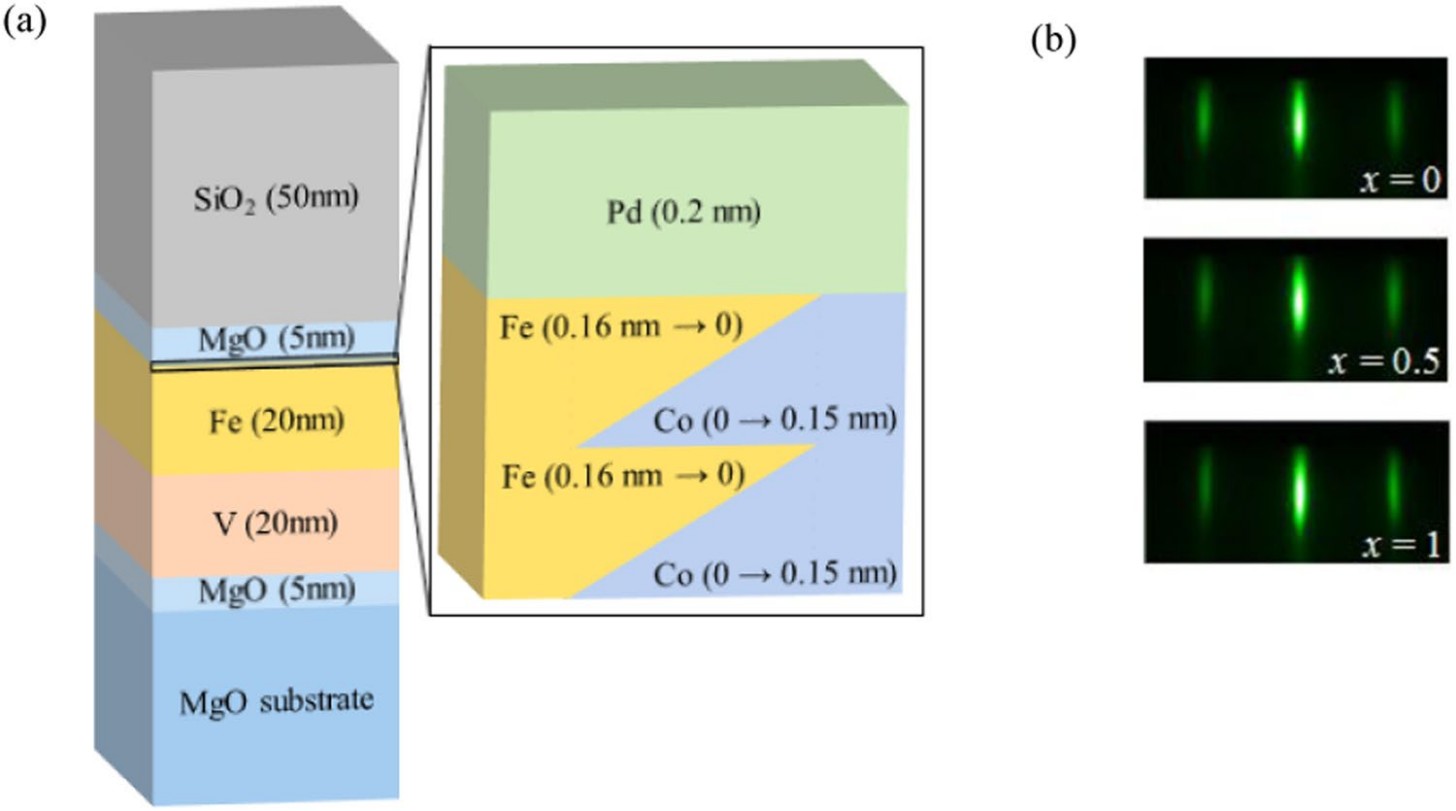
(**a**) Schematic of the film structure. (**b**) RHEED patterns of the Fe_1−*x*_Co_*x*_ surface for *x* = 0, 0.5, and 1; electron-beam//MgO [100]//Fe [110]; no change in lattice spacing was observed, which suggests a BCC-lattice formation in all regions.

Epitaxial multilayers of MgO (5 nm)/V (20 nm)/Fe (20 nm)/Fe_1−*x*_Co_*x*_ (0.3 nm)/Pd (0.2 nm)/MgO (5 nm) were deposited on a face-centered-cubic-(FCC)-MgO(001) substrate using electron-beam deposition under an ultrahigh vacuum. An ultrathin Fe_1−*x*_Co_*x*_ layer was prepared by an alternate deposition of Fe one monolayer in wedge shape and one monolayer Co was in opposite wedge shape at room temperature onto the body-centered-cubic-(BCC)-Fe-(001) layer, which was, prior to the deposition, annealed at 250 °C and cooled down to room temperature. After that, similarly, we deposited again Fe one monolayer in wedge shape and one monolayer Co was in opposite wedge shape. Schematic diagram of deposited film is shown in Fig. 1(a). The surface crystal structure of the Fe_1−*x*_Co_*x*_ layer was characterized *in situ* by RHEED, as shown in Fig. 1(b). Similar patterns were obtained for all three regions (i.e., *x* = 0, 0.5, 1). This indicated that the crystal structure was independent of the Co fraction (*x*). A 0.2-nm-thick Pd and 5-nm-thick MgO layers were then deposited on the Fe_1−*x*_Co_*x*_ layer at room temperature without annealing. Subsequently, a 50-nm-thick SiO_2_ layer was added as an additional insulating layer by sputtering at room temperature.

**Figure 2:**
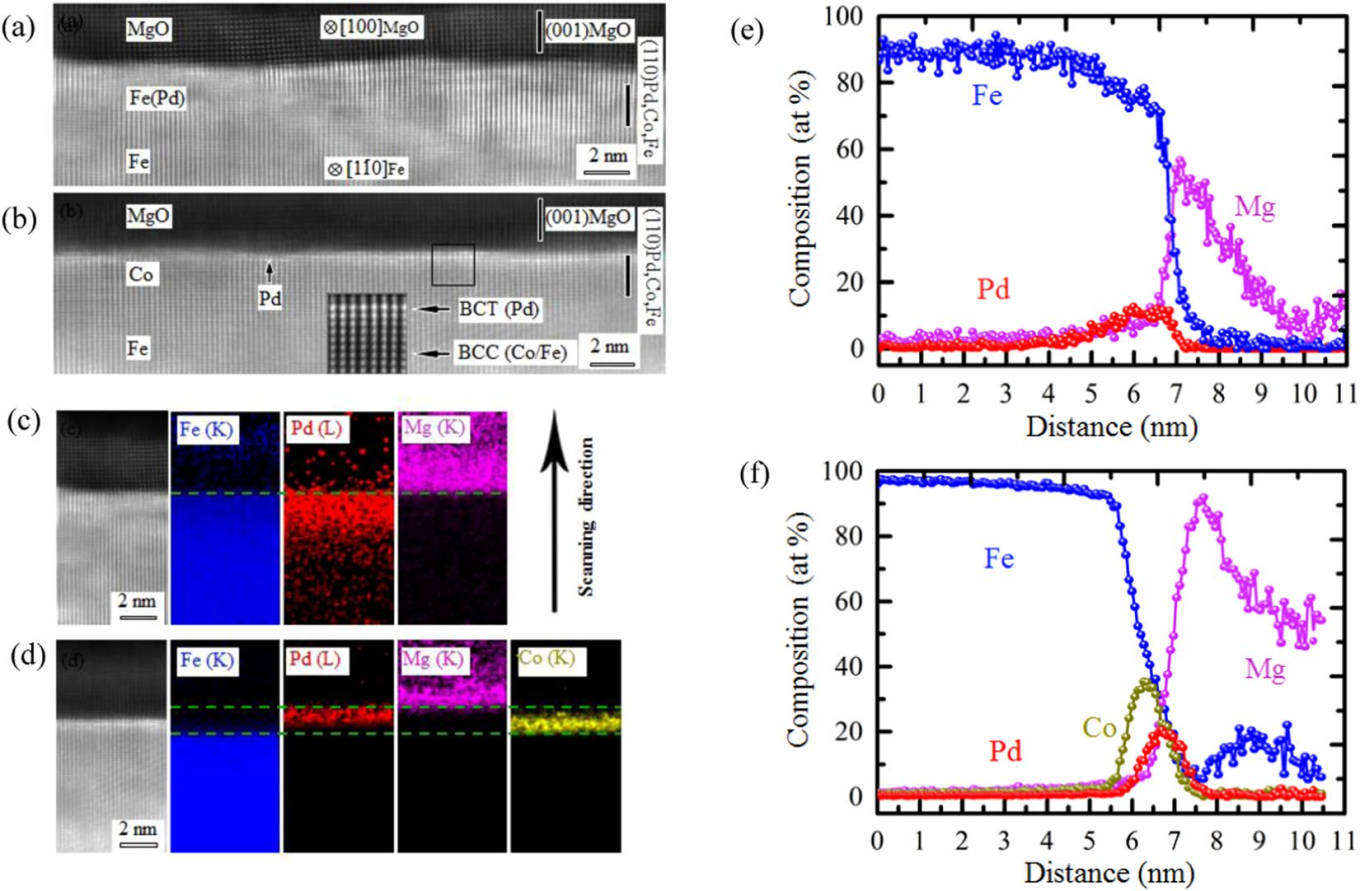
Wide-area STEM images: (**a**) area without Co content (*x* = 0), where a rough interface with MgO is observed; (**b**) pure Co region (*x* = 1), where a smoother interface with MgO is observed. At the interface, Pd atoms are observed as brighter spots. (**c**) EDS images for the *x* = 0 region. The diffusion of Pd into the Fe layer can be observed. (**d**) EDS images for the *x* = 1 region. The Pd layer is well separated from the Co layer. EDS line profiles for the (**e**) *x* = 0 and (**f**) *x* = 1 regions; the Pd diffusion length in the *x* = 0 region is estimated to be approximately 2 nm.

The HAADF-STEM image of the specimen acquired from the *x* = 0 (Fe/Pd(0.2 nm)/MgO) region (Fig. 2(a)), and that acquired from the *x* = 1 (Fe/Co(0.3 nm)/Pd(0.2 nm)/MgO) region (Fig. 2(b)) show the stacking structure of the layers with atomic resolution. The *x* = 0 region exhibits a rather rough interface with the MgO layer. The roughness is caused not only by surface atomic steps, but also by a significant lattice distortion, as shown in the atomic-resolution HAADF image. In contrast, the *x* = 1 region exhibits a flatter interface. In addition, the Pd atomic columns can be observed as brighter spots at the interface with MgO. The HAADF-STEM image (Fig. 2(b)) shows that the Pd layer exhibits a body-centered-tetragonal (BCT) structure (*a* = *b* = 2.86 Å, *c* = 3.06 Å). The BCT-Pd layer exhibits a full lattice coherency with the neighboring FCC-MgO and BCC-Co/Fe layers; the orientation relationship is: (110)[001]_BCC−Co/Fe_//(110)[001]_BCT−Pd_//(001)[010]_FCC−MgO_. Figure 2(c,d) show EDS images for the *x* = 0 and *x* = 1 regions, respectively. Diffusion of Pd into the Fe layer is observed for the *x* = 0 region (Fig. 2(c)). From the line profile in Fig. 2(e), the diffusion length of Pd is estimated to be approximately 2 nm. The diffusion of Pd, which has a different atomic diameter, leads to a distortion of the crystal lattice and rough interface.

The EDS image for the *x* = 1 region (Fig. 2(d)) shows that the Pd atoms are located in-between the MgO and Co layers. Owing to the film roughness and limited resolution of EDS, we cannot easily estimate the degree of mixing between the Pd and Co layers. The EDS line profiles show line-widths of 1.2 nm and 0.7 nm of the Co and Pd layers, respectively (Fig. 2(f)). These line-widths should be larger than the actual film thicknesses owing to the limited resolution and film roughness (0.3–0.4 nm). However, the peak positions in the concentration profiles correctly indicate the positions of the centers of the films. The highest peaks of the Co and Pd distribution signals are approximately 0.19 nm apart. This indicates that the distance between the center positions of the Co and Pd layers is slightly smaller than the designed distance (0.24 nm). As the high-resolution HAADF-STEM images show distinct Pd atomic columns at the interface, this small deviation can originate from the surface roughness caused by atomic steps and small physical mixing at the interface.

**Figure 3:**
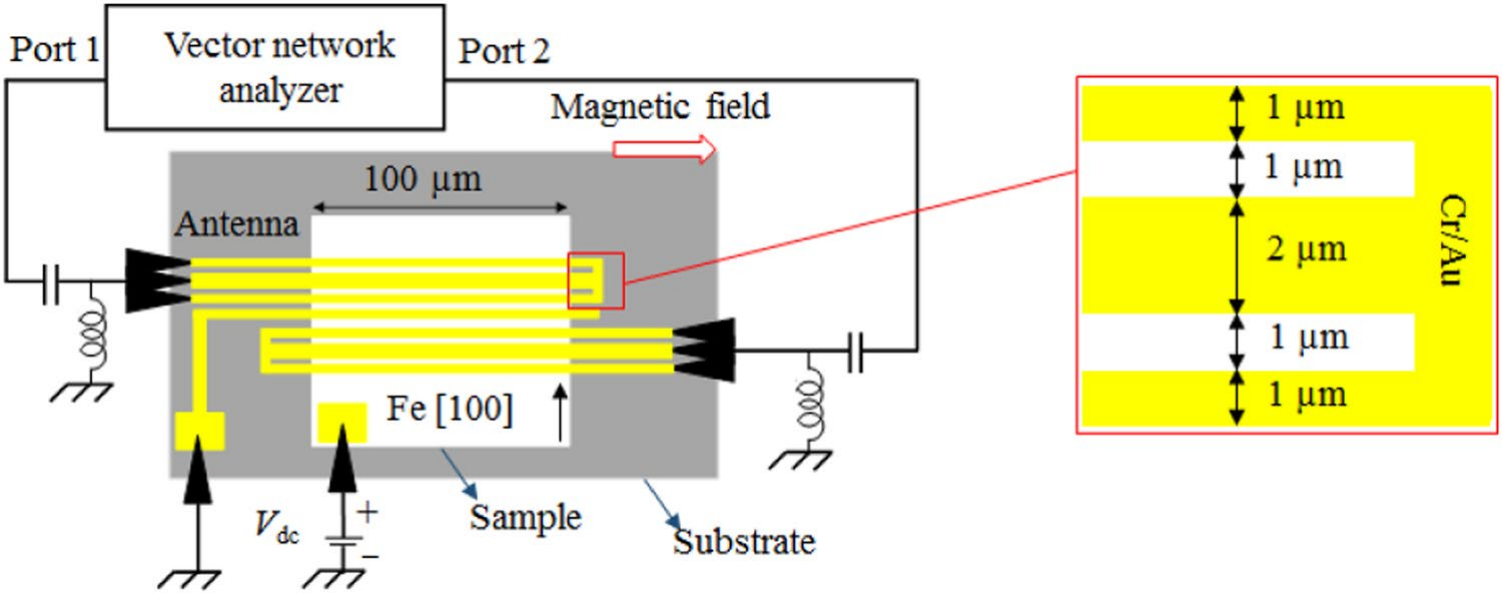
Schematics of the device structure and measurement setup. The Fe [100] direction is represented by the black arrow. The yellow color represents the antenna and gated contact pad. The ground-signal-ground (GSG) probes are shown in black. A DC voltage (*V*_dc_) is applied on the sample. A 2-µm-wide gate electrode is inserted between the antennas. The inset shows a magnified view of the antenna.

**Figure 4:**
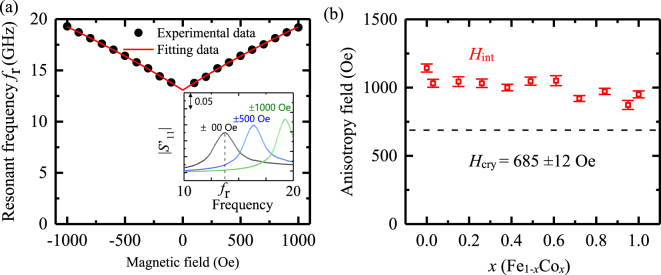
(**a**) Typical resonant frequencies of MSSWs of Fe/Fe_1−*x*_Co_*x*_/Pd/MgO with *x* = 1 (Fe/Co(0.3 nm)/Pd(0.2 nm)/MgO) under an in-plane magnetic field (*H*_ext_). The black dots and red line represent the experimental and fitting data using Eq. 1, respectively. (**b**) Fourfold crystalline anisotropy field *H*_cry_ (dashed line) and interfacial magnetic anisotropy *H*_int_ (red rectangles); the error bars represent the standard deviation of the anisotropy field obtained by the root-mean-square method. The value of *x* represents the fraction of Co in Fe_1−*x*_Co_*x*_.

The film was patterned into rectangles with dimensions of 100 × 400 µm^2^. The longer edge of the rectangle is parallel to both Fe [100] and MgO [110] directions. Microsized antennas and intermediate gate were fabricated with Cr (5 nm)/Au (200 nm) by a conventional microfabrication technique using electron-beam lithography and lift-off methods on the rectangular pattern. The antennas (short-circuited coplanar wave-guides) were designed parallel to the shorter edge of the rectangular pattern. The signal line, ground line, and gap have thicknesses of 1, 2, and 1 µm, respectively. The antennas are 10 µm apart; a 2-µm-wide gate electrode is positioned between them. The antenna excites and detects spin-waves with a wavenumber of 1.2 µm^−1^. A contact pad is fabricated by etching the rectangular pattern down to the Fe layer. A DC bias voltage (*V*_dc_) is applied between the contact pad and intermediate gate. A bias-tee was inserted between a vector network analyzer (VNA) and antenna. Figure 3 shows schematics of the spin-wave device and measurement setup. We applied a static magnetic field in the in-plane direction (*H*_ext_). Magnetostatic surface spin-waves (MSSWs) were excited by applying a radio-frequency (RF) signal of −15 dBm (32 µW). The scattering (*S*) parameter was measured using the VNA. The resonant frequency of the MSSW was obtained from the $|{{S}^{^{\prime} }}_{11}|$ $[{{S}^{^{\prime} }}_{11}={S}_{11}({H}_{ext})-{S}_{11}({\rm{2700Oe}})]$ signal (Fig. 4(a)). The $|{{S}^{^{\prime} }}_{11}|$ signal for the *x* = 1 (Fe/Co(0.3 nm)/Pd(0.2 nm)/MgO) layer, measured using the VNA, is shown in the inset of Fig. 4(a). The ${S}_{11}({\rm{2700Oe}})$ signal is considered a background signal. Similarly, $|{{S}^{^{\prime} }}_{11}|$ signals were measured in Fe_1−*x*_Co_*x*_/Pd for various values of *x*. The resonant frequency^10^ is

1 $$f=-\,\frac{\gamma }{2\pi }\sqrt{(|{H}_{{\rm{ext}}}|+{H}_{{\rm{cry}}})(|{H}_{{\rm{ext}}}|+{M}_{{\rm{S}}}+{H}_{{\rm{cry}}}-{H}_{{\rm{int}}})+\frac{{M}_{{\rm{S}}}}{4}({M}_{{\rm{S}}}-{H}_{{\rm{int}}})(1-\exp (-2|k|{t}_{{\rm{Fe}}}))}$$

where *γ*/2π (−2.94 × 10^10^ T^−1^ s^−1^) is the gyromagnetic ratio, *µ*_0_*M*_s_ (2.16 T) is the saturation magnetization, *k* (1.2 µm^−1^) is the wavenumber (estimated from the antenna design), and *t*_Fe_ (20 nm) is the thickness of the bulk Fe layer; *H*_int_ represents the interfacial magnetic anisotropy field and *H*_cry_ is the fourfold crystal anisotropy field of the Fe/Fe_1−*x*_Co_*x*_/Pd system. The values of *H*_cry_ and *H*_int_ are estimated using the least mean-square-error method, as shown in Fig. 4(b). The value of *H*_cry_ is 685 ± 12 Oe, which is similar to the fourfold anisotropy field of bulk Fe. The interfacial anisotropy field *H*_int_ varies throughout the sample up to approximately 30%, as a function of the Co fraction *x* in the Fe/Fe_1−*x*_Co_*x*_/Pd/MgO system.

**Figure 5:**
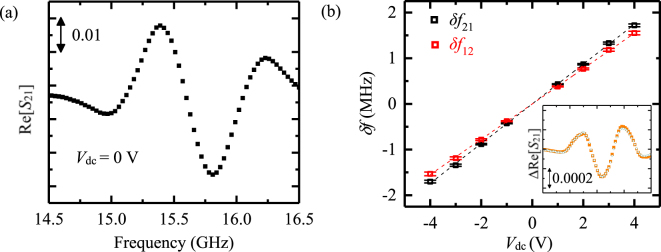
(**a**) Typical propagating spin-wave signal, which is the real part of the MSSW signal without any bias voltage. (**b**) Voltage-induced resonant-frequency shift of the propagating spin-waves (*δf*). The black (red) rectangular dots represent the frequency change of Re[*S*_21_] (Re[*S*_12_]). The black and red lines are the fitting lines for the frequency shifts *δf*_21_ and *δf*_12_, respectively. The error bars represent the standard deviation of the frequency shift obtained from the root-mean-square-error method. The inset shows the experimental (yellow solid rectangles) and fitting (green open diamond) data. The *x*-axis of the inset is the frequency axis, from 14.5 GHz to 16.5 GHz. All data in this figure correspond to the Fe_1−*x*_Co_*x*_/Pd/MgO sample with *x* = 1, i.e., Fe/Co(0.3 nm)/Pd(0.2 nm)/MgO.

Figure 5(a) shows the real part of the propagating spin-wave ($\mathrm{Re}[{S}_{21}]$) at *H*_ext_ = 400 Oe for *x* = 1 (Fe/Co(0.3 nm)/Pd(0.2 nm)/MgO). The DC bias voltage (*V*_dc_) shifts Re[*S*_21_ (0 V)] by ΔRe[*S*_21_] = Re[*S*_21_(*V*_dc_)] − Re[*S*_21_(0 V)]. It can be easily fitted as: ${\rm{\Delta }}\mathrm{Re}[{S}_{21}]=-\,\delta {f}_{21}\times d\mathrm{Re}[{S}_{21}(0\,{\rm{V}})]/df$; $\delta {f}_{21}$ represents the voltage-induced MSSW frequency-shift, which can be obtained using the least mean-square-error method. The experimental and fitted ${\rm{\Delta }}\mathrm{Re}[{S}_{21}(4\,{\rm{V}})]$ for *x* = 1 (Fe/Co(0.3 nm)/Pd(0.2 nm)/MgO) are shown in the inset in Fig. 5(b). Similarly, we estimated $\delta {f}_{12}$ from the $\mathrm{Re}[{S}_{12}]$ signal. The dependences of $\delta {f}_{12}$ and $\delta {f}_{21}$ as a function of the bias voltage are shown in Fig. 5(b) for the *x* = 1 (Co/Pd/MgO) region. The slopes of the linear fits in Fig. 5(b) represent the voltage-induced MSSW frequency-shifts per volt *δf*_12_/*V*_dc_ and *δf*_21_/*V*_dc_ for the *x* = 1 (Fe/Co(0.3 nm)/Pd(0.2 nm)/MgO) region. Similarly, *δf*_12_/*V*_dc_ and *δf*_21_/*V*_dc_ are determined for the Fe_1−*x*_Co_*x*_/Pd/MgO alloy. The symmetry term (*δf*_12_ + *δf*_21_)/2*V*_dc_ is correlated with the VCMA (Eq. 2)^10^.

2 $${\rm{VCMA}}=\frac{-f\,(\frac{\delta {f}_{21}+\delta {f}_{12}}{2{V}_{{\rm{dc}}}})\,{M}_{{\rm{S}}}\,{t}_{{\rm{Fe}}}}{\,{(\frac{\gamma }{2\pi })}^{2}(|{H}_{{\rm{ext}}}|+{H}_{{\rm{cry}}}+\frac{{M}_{{\rm{S}}}}{4}(1-{e}^{-2|k|{t}_{{\rm{Fe}}}}))\,{E}_{{\rm{MgO}}}}$$

where *E*_MgO_ is the perpendicular electric field in the 5-nm-thick MgO layer. We modelled the sample as two parallel-plate capacitors (50-nm-thick SiO_2_ (*ε* = 3.9) and 5-nm-thick MgO (*ɛ* = 9.6)).

**Figure 6:**
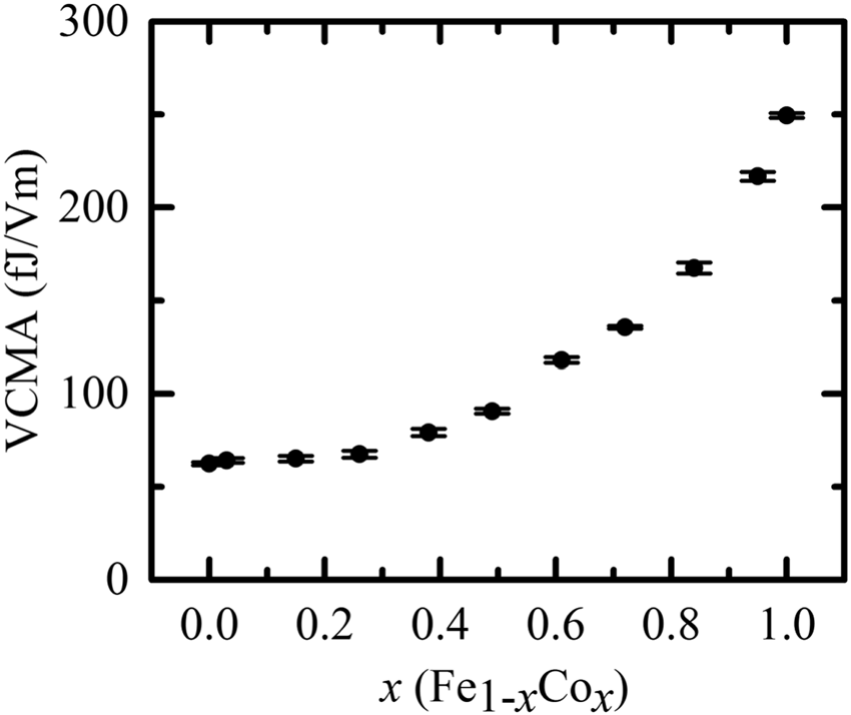
VCMA in the Fe_1−*x*_Co_*x*_/Pd/MgO system.

We observed the directional symmetry of the MSSW frequency-shift *δf*. The MSSW frequency-shift *δf* is attributed to the VCMA. The *δf*_12_ and *δf*_21_ terms linearly depend on the voltage, as shown in Fig. 5(b), without hysteresis or aging effects, as the behavior is not attributed to magneto-ionic control^23–25^. The VCMA in the Fe_1−*x*_Co_*x*_/Pd/MgO system is estimated in Fig. 6. It shows that the VCMA increases with the Co fraction *x* in the alloy. We achieved a VCMA of approximately 250 fJ/Vm at the Co/Pd/MgO region.

In addition, the Fe- and Co-composition dependence of the VCMA in the Fe_1−*x*_Co_*x*_/Pd/MgO system was studied. The VCMA increases by approximately 300% with the increase of *x* from 0 to 1, while the interfacial anisotropy energy changes by approximately 30%. These two observations suggest that the origins of VCMA and interfacial anisotropy are not the same. The STEM and EDS images show that in the *x* = 1 (Co/Pd/MgO) region, the Pd atoms are located between the MgO and Co layers, leading to a larger VCMA of 250 fJ/Vm. There are two possible origins of the large VCMA, which are the *x*-dependence of the electron occupancy in the *d-*orbital states in Fe_1−*x*_Co_*x*_ and existence of Pd atoms at the interface for *x* = 1. However, it is challenging to determine the origin of the VCMA only with the experimental results; first-principle calculations would be helpful to study the origin of the observed behavior.

In conclusion, we investigated the VCMA and *H*_int_ in the fully epitaxially grown V/Fe/Fe_1−*x*_Co_*x*_/Pd/MgO system. The VCMA increased by approximately 300% with the increase of *x* from 0 (Fe/Pd/MgO) to 1 (Co/Pd/MgO); however, *H*_int_ varied throughout the sample up to approximately 30%. Therefore, the VCMA and interfacial anisotropy energy were not directly correlated. A high VCMA of 250 fJ/Vm was achieved in the Co/Pd/MgO region, where Pd atoms were located in-between the MgO and Co layers.

## Methods

The sample was grown using electron-beam deposition under an ultrahigh vacuum. The MgO substrate was annealed at 300 °C for 30 min and 800 °C for 10 min. The MgO (5 nm) and V (20 nm) layers were epitaxially grown consecutively at 150 °C. The sample was then annealed at 500 °C for 30 min. The Fe (20 nm) layer was epitaxially grown at room temperature, and then the sample was annealed at 250 °C for 15 min. The Fe_1−*x*_Co_*x*_ (0.3 nm)/Pd (0.2 nm)/MgO (5 nm) layers were epitaxially grown at room temperature. The RHEED image was recorded *in-situ*. The SiO_2_ (50 nm) layer was then deposited in the sputtering system. The HAADF-STEM and EDS images were acquired from the deposited sample. The film was patterned into rectangles with dimensions of 100 × 400 µm^2^. The longer edge of the rectangle was parallel to both Fe [100] and MgO [110] directions. Microsized antennas and intermediate gate were fabricated with Cr (5 nm)/Au (200 nm) by a conventional microfabrication technique using electron-beam lithography and lift-off methods on the rectangular pattern. The antennas (short-circuited coplanar wave-guides) were designed parallel to the shorter edge of the rectangular pattern. The signal line, ground line, and gap have thicknesses of 1, 2, and 1 µm, respectively. The antennas are 10 µm apart and a 2-µm-wide gate electrode is positioned between them. The antenna excites and detects spin-waves with a wavenumber of 1.2 µm^−1^. A contact pad is fabricated by etching the rectangular pattern down to the Fe layer. A DC bias voltage (*V*_dc_) is applied between the contact pad and intermediate gate. A bias-tee was inserted between the VNA and antenna. Figure 3 shows schematics of the spin-wave device and measurement setup. We applied a static magnetic field in the in-plane direction (*H*_ext_). MSSWs were excited by applying an RF signal of −15 dBm (32 µW). The scattering (*S*) parameter was measured using the VNA.
